# Single-Cell Measurements and Modeling and Computation of Decision-Making Errors in a Molecular Signaling System with Two Output Molecules

**DOI:** 10.3390/biology12121461

**Published:** 2023-11-23

**Authors:** Ali Emadi, Tomasz Lipniacki, Andre Levchenko, Ali Abdi

**Affiliations:** 1 Center for Wireless Information Processing, Department of Electrical and Computer Engineering, New Jersey Institute of Technology, 323 King Blvd, Newark, NJ 07102, USA; ae378@njit.edu; 2 Institute of Fundamental Technological Research, Polish Academy of Sciences, Pawinskiego 5B, 02-106 Warsaw, Poland; tlipnia@ippt.pan.pl; 3 Yale Systems Biology Institute, Yale University, New Haven, CT 06520, USA; 4 Department of Biomedical Engineering, Yale University, New Haven, CT 06511, USA; 5 Department of Biological Sciences, New Jersey Institute of Technology, 323 King Blvd, Newark, NJ 07102, USA

**Keywords:** cell decision making, decision theory, molecular signaling systems, signal transduction noise, cellular decision error probabilities

## Abstract

**Simple Summary:**

Cells continually sense and receive signals from the environment and respond accordingly. Due to biological noise, however, the response is not always as expected. Such a response can induce a different cell fate and may disrupt some cellular functions. In the presence of noise, cells may either mistakenly perceive non-existent signals and act accordingly, or may ignore the actual signals and do nothing. We label these two as false alarm and signal miss events, respectively. In this paper, we consider an important signaling system with one input and two outputs to show how the likelihood of false alarm and signal miss events can be computed, using the experimentally measured joint response of the two outputs of the signaling system. The two system outputs are the nuclear factor κB (NFκB) and the activating transcription factor-2 (ATF-2), whereas the system input is the tumor necrosis factor (TNF). These molecules are highly involved in essential processes such as cell survival, cell death, and viral replication. The introduced methodology and the measured false alarm and miss probabilities using experimental data can model complex cellular decision-making processes and provide insight into how they may contribute to the development of some pathological conditions.

**Abstract:**

A cell constantly receives signals and takes different fates accordingly. Given the uncertainty rendered by signal transduction noise, a cell may incorrectly perceive these signals. It may mistakenly behave as if there is a signal, although there is none, or may miss the presence of a signal that actually exists. In this paper, we consider a signaling system with two outputs, and introduce and develop methods to model and compute key cell decision-making parameters based on the two outputs and in response to the input signal. In the considered system, the tumor necrosis factor (TNF) regulates the two transcription factors, the nuclear factor κB (NFκB) and the activating transcription factor-2 (ATF-2). These two system outputs are involved in important physiological functions such as cell death and survival, viral replication, and pathological conditions, such as autoimmune diseases and different types of cancer. Using the introduced methods, we compute and show what the decision thresholds are, based on the single-cell measured concentration levels of NFκB and ATF-2. We also define and compute the decision error probabilities, i.e., false alarm and miss probabilities, based on the concentration levels of the two outputs. By considering the joint response of the two outputs of the signaling system, one can learn more about complex cellular decision-making processes, the corresponding decision error rates, and their possible involvement in the development of some pathological conditions.

## 1. Introduction

A cell has to recognize and respond to environmental variations and changes and biological noise [1,2,3]. Cell fate can change based on the strength or concentration level of extracellular stimuli or input signals. Signal transduction noise can disturb the input signals such that the cell becomes unable to correctly sense the precise concentration of different input signals and subsequently cannot appropriately respond [4,5,6,7]. Cellular decision making has been extensively studied from various angles [7,8,9,10,11,12]. Due to the random nature of signal transduction noise, a cellular decision is probabilistic to some extent [5,7]. To quantify and characterize the cell decision-making processes while incorporating their probabilistic nature, we consider a statistical signal processing approach [13]. This method is built on decision theory and statistical signal processing concepts to obtain optimal decision thresholds and erroneous cell decision probabilities using single-cell data [13]. Such a framework aims to measure the ability of the system to correctly decide on an input signal. This quantitative and probabilistic approach can also be used to characterize stochastic signaling mechanisms and phenotype induction in the context of genetic diseases [14]. One may also expand this approach to study intercellular processes together with intracellular molecular networks [15].

**Figure 1:**
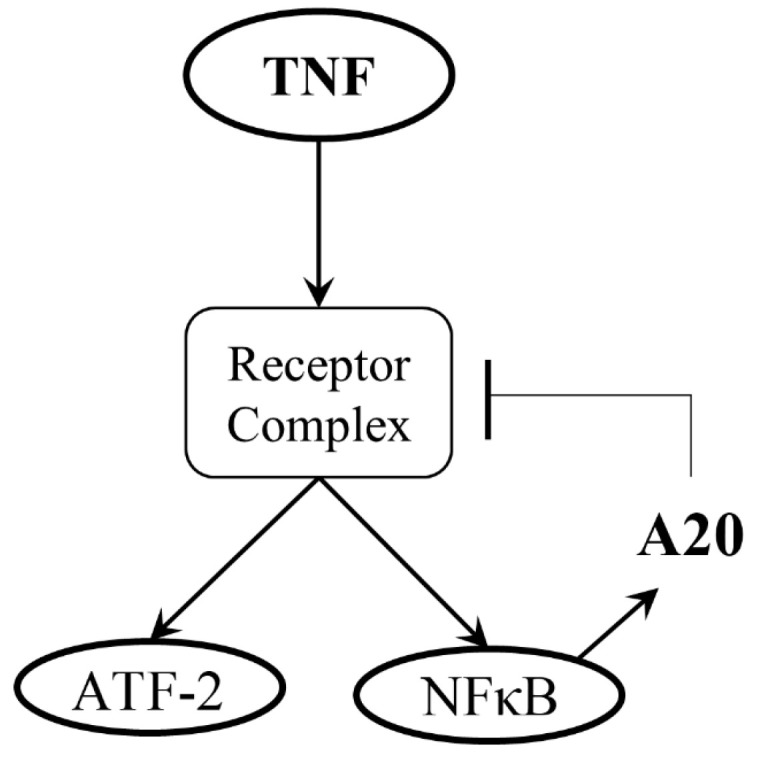
The two-output TNF—NFκB/ATF-2 signaling system.

In this paper, we consider a two-output signaling system (Figure 1) in which the tumor necrosis factor (TNF) regulates the two transcription factors, the nuclear factor κB (NFκB) and the activating transcription factor-2 (ATF-2) [5]. TNF can mediate anti-apoptotic and pro-apoptotic signals, and may also trigger necroptosis as a form of pro-inflammatory cell death [16,17]. Moreover, TNF, a key antiviral cytokine, can significantly damage healthy tissues [18,19]. It has also been shown that it can regulate a speed–accuracy tradeoff in the context of cell death decisions [20]. NFκB, as the nuclear effector of a signaling pathway, can respond to many environmental triggers across various cell types [21,22]. Furthermore, TNF activates NFκB, leading to its nuclear translocation. It has been shown that NFκB, an essential gene regulator, can respond to various doses of TNF, and its activation may prevent a cell from apoptosis [22,23,24]. NFκB is highly involved in a wide range of pathological and physiological processes, such as inflammation, adaptive immune responses, innate immune responses, secondary lymphoid organ development, autoimmune diseases, and various types of cancer [25,26,27]. The A20 (Figure 1) mediates negative inhibitory feedback on the system input [5,28]. Due to this negative feedback, NFκB level may experience a reduction. With regard to the other system output, ATF-2, we note that TNF is able to activate the c-Jun N-terminal kinase (JNK) pathway and stimulate phosphorylated ATF-2 [5]. The ATF/CREB family has important physiological functions and represents a large group of basic-region leucine zipper (bZIP) transcription factors (TFs) [29]. ATFs act as heterodimers or homodimers with different bZIP transcription factors. The family includes ATF-1, ATF-2, ATF-3, ATF-4, ATF-5, ATF-6, and ATF-7, whose abilities are diversely associated with the cellular processes that they regulate [29].

In this paper, we aim to show how the statistical decision-making framework developed for signaling systems that have only one output [13] can be extended to systems with two or more outputs. More specifically, in the TNF system (Figure 1), we compute and show what the decision thresholds are when measurements of the concentration levels of the two outputs, NFκB and ATF-2, are considered. We also define and compute decision error probabilities based on the two-output measurements and compare them with single-output decisions. By considering the joint response from the two outputs of the system, we intend to take one further step toward understanding cell decision-making processes and the associated decision error probabilities.

The rest of this paper is organized as follows. First, detailed explanations of the computational and analysis methods are discussed. Then, the single-cell experimental data of the TNF—NFκB/ATF-2 signaling system are introduced. Afterward, the two-output system data and the associated decision threshold boundaries are computed and presented in graphical form, followed by presenting and discussing various computed decision error probability results. At the end, some concluding results are presented.

## 2. Materials and Methods

In this paper, cellular decision making in the TNF—NFκB/ATF-2 signaling system is considered as the following hypothesis testing problem:

(1) $$\left\{\begin{array}{c} \begin{array}{l} H_{0}:\;\;\mathrm{TNF}\;\;\mathrm{level}\;\;\mathrm{is}\;\;\mathrm{low},\; \end{array}\\ H_{1}:\;\;\mathrm{TNF}\;\;\mathrm{level}\;\;\mathrm{is}\;\;\mathrm{high}. \end{array}\right.$$

A cell may make each of these two mistakes due to signal transduction noise: deciding that TNF is high at the input of the system while it is actually low—declaring H_1_ while H_0_ is true—or deciding that TNF is low although, in fact, it is high—declaring H_0_ when H_1_ is true. These are false alarm and miss incorrect decisions, respectively, with the following probabilities:

(2) $$\begin{array}{l} P_{\mathrm{FA}}=P(\mathrm{deciding}\;\;H_{1}{|H}_{0}),\\ P_{M}\;=P(\mathrm{deciding}\;\;H_{0}{|H}_{1}). \end{array}$$

Additionally, the overall error probability *P*_E_ of making decisions is a weighted summation of *P*_FA_ and *P*_M_:

(3) $$P_{E}=P(H_{0})P_{\mathrm{FA}}+P(H_{1})P_{M},$$

where *P*(H_0_) and *P*(H_1_) represent the prior probabilities of the hypotheses H_0_ and H_1_, respectively.

The optimal decision-making approach minimizes *P*_E_ [30,31]. To understand how such decisions are made, suppose **z** is the *N*-element vector of observed variables or data (in our case, we have *N* = 2, and the components of **z** represent NFκB and ATF-2). Let *p*(**z**|H_0_) and *p*(**z**|H_1_) be the conditional probability density functions (PDFs) of **z** under the hypotheses H_0_ and H_1_, respectively. The optimal decision rule is derived from the maximum likelihood principle [31], which chooses the hypothesis with the highest likelihood of occurrence as the best—optimal—decision. More specifically, the optimal decision rule compares the conditional likelihood ratio *L*(**z**) = *p*(**z**|H_1_)/*p*(**z**|H_0_) with the likelihood threshold of *γ* = *P*(H_0_)/*P*(H_1_) and decides H_1_ if *L*(**z**) > *γ*, or decides H_0_ otherwise. In general, *L*(**z**) = *γ* represents the optimal decision threshold hypersurface, and for *N* = 2, it represents the optimal decision threshold curve (DTC).

To evaluate the performance of this optimal decision rule, false alarm and miss probabilities [31] need to be computed using the following *N*-variate integrals:

(4) $$\begin{array}{l} P_{\mathrm{FA}}=\int \ldots \int p(z|H_{0})\;\;\;dz,\\ \;\;{\;}_{\{z\in \;\mathrm{False}\;\mathrm{Alarm}\;\mathrm{Region}\}} \end{array}$$

(5) $$\begin{array}{l} P_{M}=\int \ldots \int p(z|H_{1})\;\;\;dz.\\ \;\;{\;}_{\{z\in \;\mathrm{Miss}\;\mathrm{Region}\}} \end{array}$$

As discussed later, Gaussian PDFs can represent the data. An *N*-variate Gaussian PDF for **z** under the *i*-th hypothesis H*_i_* can be written as follows [31,32]:

(6) $$p(z|H_{i})=\frac{1}{{\left(2\pi \right)}^{N/2}|\Sigma_{i}{|}^{1/2}}\exp\left[-\frac{1}{2}{\left(z-\mu_{i}\right)}^{T}\Sigma_{i}^{-1}(z-\mu_{i})\right],\;\;\;\;i=0,1\;\;.$$

In the above equation, $\mu_{i}$ and $\Sigma_{i}$ are the mean vector and the covariance matrix of **z** under H*_i_*, respectively; ${|\Sigma }_{i}|$ and $\Sigma_{i}^{-1}$ represent the determinant and the inverse of the matrix $\Sigma_{i}\;$, respectively; and *^T^* denotes the transpose operation.

### 2.1. Using the Likelihood Ratio to Compute the Optimal Decision Thresholds and the Decision Error Probabilities for the TNF—NFκB/ATF-2 System

To compute the optimal decision thresholds and the decision error probabilities using the likelihood ratio, first, we present the univariate methods that have simpler equations, followed by the bivariate methods.

#### 2.1.1. Univariate Decision Analysis

We use the nuclear NFκB and ATF-2 concentrations of thousands of cells that are exposed to TNF concentrations of 0.013, 0.082, 3.2, and 50 ng/mL after 30 min and 4 h [5]. For the univariate decision analysis, let us define *x* = ln(Nuclear NFκB), where ln is the natural logarithm. An examination of the data reveals that a Gaussian PDF can be used to model the *x* variable:

(7) $$p(x|H_{i})=\frac{1}{{\left(2\pi \sigma_{i}^{2}\right)}^{1/2}}\exp\left[\frac{-{\left(x-\mu_{i}\right)}^{2}}{2\sigma_{i}^{2}}\right],\;\;\;\;i=0,1\;\;,$$

where $\mu_{i}$ and $\sigma_{i}^{2}$ are the mean and the variance under the *i*-th hypothesis H*_i_*, respectively. The TNF level under H_0_ is 0.013 ng/mL, while under H_1_, it is 0.082, 3.2, or 50 ng/mL.

The optimal likelihood-based decision rule decides H_1_ if:

(8) $$L(x)=\frac{p(x|H_{1})}{p(x|H_{0})}\;\;\;>1\;\;,$$

for equi-probable hypotheses. To obtain the optimal decision threshold for *x*, the following *p*(*x*|H_1_) = *p*(*x*|H_0_) equation needs to be solved for *x*:

(9) $$\frac{1}{{\left(2\pi \sigma_{1}^{2}\right)}^{1/2}}\exp\left[\frac{-{\left(x-\mu_{1}\right)}^{2}}{2\sigma_{1}^{2}}\right]=\frac{1}{{\left(2\pi \sigma_{0}^{2}\right)}^{1/2}}\exp\left[\frac{-{\left(x-\mu_{0}\right)}^{2}}{2\sigma_{0}^{2}}\right]\;.$$

After algebraic simplification of Equation (9), the following quadratic equation is obtained:

(10) $$(\sigma_{1}^{-2}-\sigma_{0}^{-2})x^{2}-2(\mu_{1}\sigma_{1}^{-2}-\mu_{0}\sigma_{0}^{-2})x+\mu_{1}{}^{2}\sigma_{1}^{-2}-\mu_{0}{}^{2}\sigma_{0}^{-2}+\ln(\sigma_{1}^{2}/\sigma_{0}^{2})=0\;\;,$$

By solving this equation numerically, the optimal decision threshold $x_{th}$ can be computed.

The false alarm and miss probabilities can be computed using the following formulas [13]:

(11) $$P_{\mathrm{FA}}=\int_{x_{\mathrm{th}}}^{\infty }p(x|H_{0})\;\;dx\;\;\;=Q\left(\frac{x_{\mathrm{th}}-\mu_{0}}{\sigma_{0}}\right),\;\;\;\;\;\;$$

(12) $$P_{M}=\int_{-\infty }^{x_{\mathrm{th}}}p(x|H_{1})\;\;dx\;\;\;=Q\left(\frac{\mu_{1}-x_{\mathrm{th}}}{\sigma_{1}}\right),$$

where the *Q* function is defined below:

(13) $$Q(\eta )={\left(2\pi \right)}^{-1/2}\;\int_{\eta }^{\infty }\exp(-u^{2}/2)\;\;du\;.$$

By defining *y* = ln(Nuclear ATF-2), all the above equations and results can be used, after replacing *x* in there with *y*.

#### 2.1.2. Bivariate Decision Analysis

For the bivariate decision analysis, we consider *x* = ln(Nuclear NFκB) and *y* = ln(Nuclear ATF-2), and define the two-element vector ${z=[x\;y]}^{T}$. Upon substituting *N* = 2 in Equation (6), the following bivariate PDF can be written for **z**:

(14) $$p(z|H_{i})=\frac{1}{2\pi |\Sigma_{i}{|}^{1/2}}\exp\left[-\frac{1}{2}{\left(z-\mu_{i}\right)}^{T}\Sigma_{i}^{-1}(z-\mu_{i})\right],\;\;\;\;i=0,1.$$

Here, $\mu_{i}$ and $\Sigma_{i}$ are the mean vector and the covariance matrix under the *i*-th hypothesis H*_i_*, respectively:

(15) $$\mu_{i}=\left[\begin{array}{c} \mu_{x,i}\\ \mu_{y,i} \end{array}\right],\;\;\;\Sigma_{i}=\left[\begin{array}{cc} \sigma_{x,i}^{2} & \rho_{i}\sigma_{x,i}^{}\sigma_{y,i}^{}\\ \rho_{i}\sigma_{x,i}^{}\sigma_{y,i}^{} & \sigma_{y,i}^{2} \end{array}\right],\;\;\;\;\;i=0,1\;\;\;,$$

where $\rho_{i}$ is the correlation coefficient between *x* and *y* under the *i*-th hypothesis H*_i_*.

The optimal likelihood-based decision rule decides H_1_ if

(16) $$L(z)=\frac{p(z|H_{1})}{p(z|H_{0})}\;\;\;>1\;\;.$$

To find the optimal DTC in the *x*-*y* plane, the following *p*(**z**|H_1_) = *p*(**z**|H_0_) equation needs to be solved in terms of *x* and *y*:

(17) $$\frac{1}{2\pi |\Sigma_{1}{|}^{1/2}}\exp\left[-\frac{1}{2}{\left(z-\mu_{1}\right)}^{T}\Sigma_{1}^{-1}(z-\mu_{1})\right]=\frac{1}{2\pi |\Sigma_{0}{|}^{1/2}}\exp\left[-\frac{1}{2}{\left(z-\mu_{0}\right)}^{T}\Sigma_{0}^{-1}(z-\mu_{0})\right]\;\;.$$

By taking the natural logarithm of both sides of Equation (17) and rearranging some terms, the following bivariate quadratic equation is obtained:

(18) $$z^{T}(\Sigma_{1}^{-1}-\Sigma_{0}^{-1})z-2(\mu_{1}^{T}\Sigma_{1}^{-1}-\mu_{0}^{T}\Sigma_{0}^{-1})z+\mu_{1}^{T}\Sigma_{1}^{-1}\mu_{1}-\mu_{0}^{T}\Sigma_{0}^{-1}\mu_{0}+\ln\;(\frac{\left|\Sigma_{1}\right|}{\left|\Sigma_{0}\right|})=0\;\;.$$

By solving this equation numerically, the optimal DTC can be computed and graphed in the *x*-*y* plane. For *N* = 1 and when **z** includes only the one variable *x*, Equation (18) reduces to Equation (10).

The false alarm and miss probabilities can be computed using Equations (4) and (5) with *N* = 2, respectively:

(19) $$P_{\mathrm{FA}}=\iint_{\left\{\;(x,y)\in \;\mathrm{False}\;\mathrm{Alarm}\;\mathrm{Region}\right\}}p(x,y|H_{0})\;\;\;dx\;dy,$$

(20) $$P_{M}=\iint_{\left\{(x,y)\in \;\mathrm{Miss}\;\mathrm{Region}\;\right\}}p(x,y|H_{1})\;\;\;dx\;dy\;.$$

The bivariate integrals in Equations (19) and (20) are computed using Monte Carlo integration.

### 2.2. Using the Discriminant Function to Compute the Decision Error Probabilities for the TNF—NFκB/ATF-2 System

Here, we explain how to use the discriminant function [33,34] to compute the decision error probabilities, without computing multivariate integrals. The discriminant function for the *i*-th hypothesis H*_i_* is defined as follows:

(21) $$g_{i}(z)=\ln\;\;p(z|H_{i})+\ln\;\;P(H_{i}),\;\;\;\;\;i=0,1\;\;.$$

By substituting Equation (6) together with *N* = 2 in (21), we obtain

(22) $$g_{i}(z)=-\frac{1}{2}{\left(z-\mu_{i}\right)}^{T}\Sigma_{i}^{-1}(z-\mu_{i})-\ln\;\;(2\pi )-\frac{1}{2}\ln\;\;|\Sigma_{i}|+\ln\;\;P(H_{i}),\;\;\;\;i=0,1\;\;.$$

The optimal decision rule decides H_1_ if $g_{1}\left(z\right)>g_{0}\left(z\right)$.

To compute the false alarm probability *P*_FA_ using the discriminant functions and the **z** data points available under H_0_, we compare the numerical values of $g_{1}\left(z\right)$ and $g_{0}\left(z\right)$ for each **z** under H_0_, count the number of times that $g_{1}\left(z\right)>g_{0}\left(z\right)$, that is, when a false alarm event occurs, and then divide it by the number of the **z** data points available under H_0_. The miss probability *P*_M_ is similarly computed.

## 3. Results and Discussion

### 3.1. Single Cell Data of the Two-Output TNF—NFκB/ATF-2 System

The data set was obtained from 3T3-immortalized mouse embryonic fibroblasts [5]. Nuclear concentrations of NFκB and ATF-2 were measured using immunocytochemistry of thousands of mouse fibroblasts exposed to different TNF levels [5]. As explained in Section 1, decision-making performance and probabilities of the two-output system in Figure 1—in response to its input TNF signal—are investigated in this paper because of the high involvement of the NFκB and ATF-2 transcription factors in cell death and survival processes.

### 3.2. Graphical Representation of the Two-Output System Data and the Decision Thresholds

**Figure 2:**
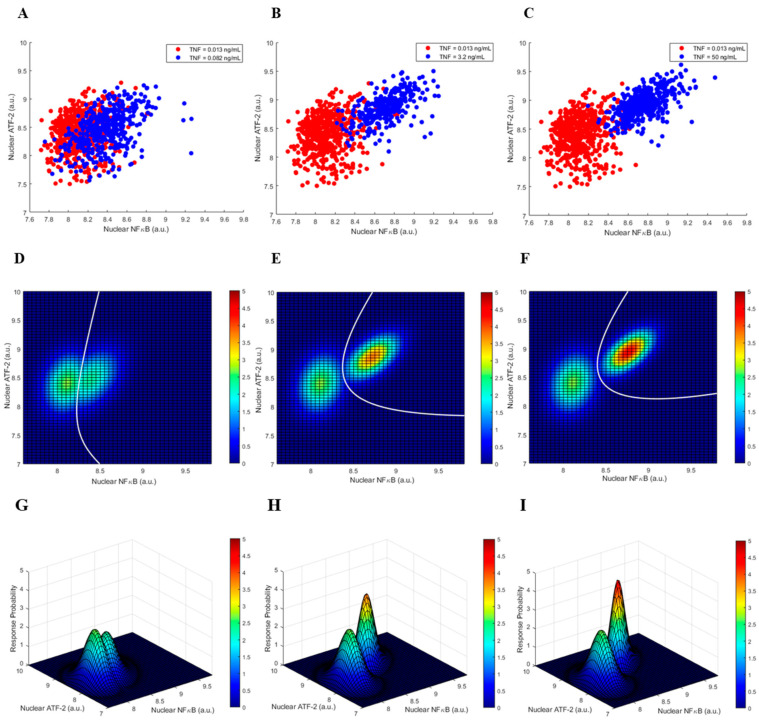
Cell responses after 30 min of TNF exposure. (**A**–**C**) Scatter plots of nuclear NFκB and ATF-2 when high TNF level is 0.082, 3.2, or 50 ng/mL, respectively. (**D**–**F**) Top-view heatmaps of bivariate Gaussian probability density functions (PDFs) for NFκB and ATF-2, when high TNF = 0.082, 3.2, or 50 ng/mL, respectively, together with the corresponding optimal decision threshold curves (DTCs) in white. (**G**–**I**) Bivariate Gaussian PDFs for NFκB and ATF-2 when high TNF = 0.082, 3.2, or 50 ng/mL, respectively (the (**D**–**F**) panels are top-view heatmaps of these bivariate Gaussian PDFs). In all cases, low TNF = 0.013 ng/mL.

To characterize and measure the decision probabilities of whether the TNF level is low or high, based upon the nuclear concentrations of NFκB and ATF-2, four TNF levels of 0.013, 0.082, 3.2, and 50 ng/mL are arbitrarily chosen, where the first is considered to be low TNF, and the last three are considered to be high TNF concentrations. Extension to deciding on more than two input signal levels, for instance, three input signal levels, is possible. Figure 2 presents some important two-output graphics of cell responses after 30 min of TNF exposure. More specifically, panels Figure 2A–C show the scatter plots of nuclear NFκB and ATF-2, where the high TNF level is 0.082, 3.2, or 50 ng/mL, respectively, compared to the fixed low TNF level of 0.013 ng/mL. The associated bivariate Gaussian probability density functions (PDFs) for nuclear NFκB and ATF-2 (see Section 2) are shown in the last row of Figure 2. Finally, panels Figure 2D–F depict the top-view heatmaps of these bivariate Gaussian PDFs, along with the corresponding optimal decision threshold curves (DTCs). An optimal DTC is a curve that divides the two-dimensional parameter space, here, the NFκB/ATF-2 plane, such that the decision error probability—defined later in Equation (3)—is minimized. Each optimal DTC is graphed by solving a quadratic equation obtained from the maximum likelihood decision-making principle (see Section 2). As an example, we see the optimal DTC in Figure 2D that divides the NFκB/ATF-2 plane such that the region on its left corresponds to the low TNF decision, and the region on its right relates to the high TNF decision. Details of how an optimal DTC is computed are provided in Section 2. Given the overlap between the two heatmap clusters, the decision error probability, based on the optimal DTC, is 0.245. As one can expect, as the high TNF level increases to 3.2 and 50 ng/mL (see Figure 2E,F), the two heatmap clusters become separated, and the decision error probability decreases to 0.05 and 0.03, respectively. These and other decision error probabilities for various scenarios are later computed and examined in Section 3.3.

**Figure 3:**
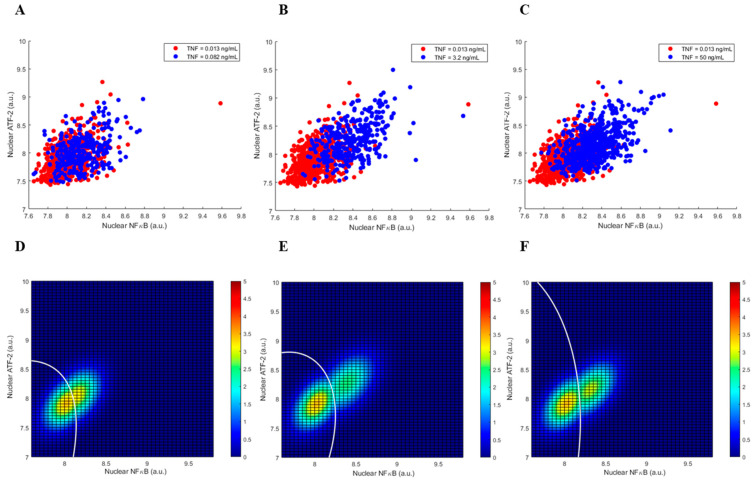
Cell responses after 4 h of TNF exposure. (**A**–**C**) Scatter plots of nuclear NFκB and ATF-2 when high TNF level is 0.082, 3.2, or 50 ng/mL, respectively. (**D**–**F**) Top-view heatmaps of bivariate Gaussian PDFs for NFκB and ATF-2, when high TNF = 0.082, 3.2, or 50 ng/mL, respectively, together with the corresponding optimal DTCs.

The two-output graphics of cell responses, after 4 h of exposure to different levels of TNF, are shown in Figure 3. We observe more overlap between the two heatmap clusters, compared to Figure 2. This can be perhaps explained by noting that the inhibitory feedback of A20 becomes active over time, which results in reductions in nuclear NFκB and ATF-2 concentrations. The associated changes in the decision error probabilities are computed and examined in Section 3.3.

### 3.3. Decision Error Probabilities of the Two-Output System

**Figure 4:**
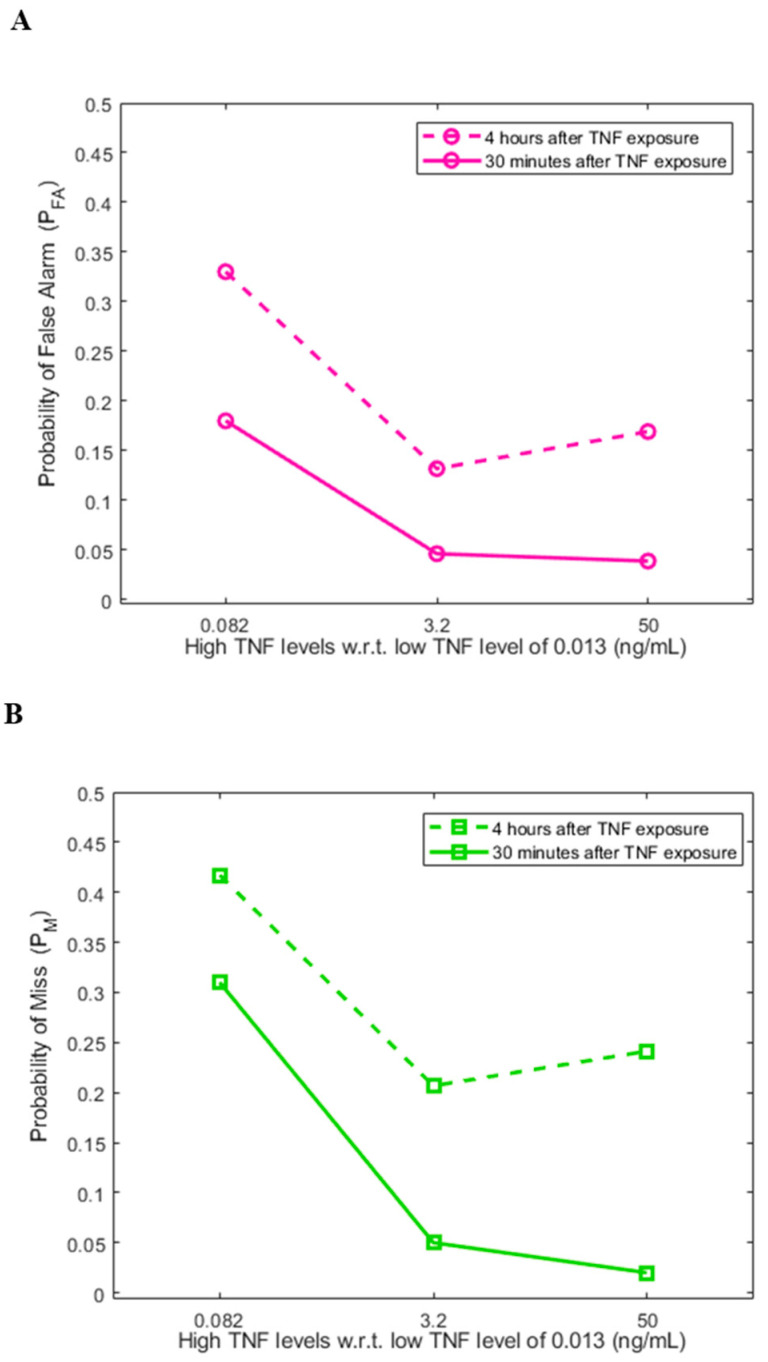
Decision error probabilities in cells based on measured nuclear NFκB and ATF-2 concentrations as the two outputs of the signaling system after 30 min and 4 h of TNF exposure. (**A**) Probabilities of false alarm. (**B**) Probabilities of miss.

Information theoretical studies of single-cell data of a TNF signaling system have demonstrated that a cell can distinguish between low and high TNF concentrations at the system input [5]. Due to the uncertainty caused by signal transduction noise, two types of incorrect decisions can be made: deciding that TNF is high while it is actually low, or deciding that TNF is low while it is actually high. These two are called false alarm and miss errors, respectively [13]. In what follows, we present (Figure 4) and discuss the computed false alarm and miss error probabilities, *P*_FA_ and *P*_M_, respectively, using measured nuclear NFκB and ATF-2 concentrations as the two outputs of the signaling system.

To graphically explain how the *P*_FA_ and *P*_M_ error probabilities are computed, we consider the high TNF level of 0.082 ng/mL in Figure 4 as an example. The computed 0.18 false alarm probability (Figure 4A, 30 min) is obtained by computing the volume under the low TNF bivariate PDF in Figure 2G that falls on the right-hand-side region of the DTC graphed in Figure 2D—A curve that divides the NFκB/ATF-2 plane into two separate decision regions. This is the false alarm region (see Section 2). Moreover, the computed 0.31 miss probability (Figure 4B, 30 min) is obtained by computing the volume under the high TNF bivariate PDF in Figure 2G that falls on the left-hand-side region of the DTC graphed in Figure 2D. This is the miss region (see Section 2). Other false alarm and miss error probabilities in Figure 4 are similarly computed.

We note a monotone decrease in both decision error probabilities at 30 min (Figure 4) as the TNF signal becomes stronger. This can be attributed to the linear structure of the pathway in the short term, when the feedback is not active yet. At 4 h, however, we do not observe a monotone decrease in the decision error probabilities as the TNF signal strength increases (Figure 4). This is perhaps because of the activation of the A20 feedback that renders a nonlinear structure for the pathway.

Another noteworthy observation is that the *P*_FA_ and *P*_M_ error probabilities are higher at 4 h (Figure 4). The two heatmap clusters exhibit more overlaps at 4 h (Figure 3) compared to 30 min (Figure 2). This can be associated with the reductions in nuclear NFκB and ATF-2 concentrations at 4 h, caused by the negative A20 feedback.

To investigate how much the importance of each individual system output is, when it comes to decision making using both outputs, we use the minimum redundancy maximum relevance (MRMR) algorithm [35] implemented in MATLAB^®^ (9.13.0.2105380 (R2022b) Update 2), originally developed for feature selection in classification problems. The MRMR algorithm finds an optimal set of features so that the redundancy in the feature set is minimized, while its relevance to the response variable is maximized. The algorithm defines relevance as the mutual information between each feature and the response variable, and measures redundancy as the mutual information among the features. By defining the mutual information quotient (MIQ) parameter as the ratio of the relevance over the redundancy of a feature, the MRMR algorithm ranks the features. It also computes an importance score for each feature using a recursive approach [36]. In our case of NFκB and ATF-2, the two outputs of the system, after finding the output with the highest rank, the algorithm assigns the corresponding relevance value as the importance score for the highest-ranked output. The importance score of the second-ranked output is the importance score of the first-ranked output multiplied by the ratio of the first-ranked output’s MIQ over the second-ranked output’s MIQ. Further details are provided in [36].

Now, we compute the importance scores for NFκB and ATF-2 (Table 1) to quantify their importance in terms of their ability to render a decision on the status of the input TNF signal. Given the lower scores of ATF-2, one may say that ATF-2 possibly plays a smaller role in the decision making. This is confirmed by computing and comparing the univariate decision error probabilities with the bivariate probabilities. For example, for the high TNF level of 0.082 ng/mL and 30 min data, we have the univariate miss probabilities of *P*_M_(NFκB) = 0.32 and *P*_M_(ATF-2) = 0.44, and the bivariate miss probability of *P*_M_(NFκB & ATF-2) = 0.31. We note that the bivariate probability is closer to the univariate probability rendered by NFκB only. For the same high TNF level and 4 h data, we observe the same pattern—the univariate miss probabilities are *P*_M_(NFκB) = 0.41 and *P*_M_(ATF-2) = 0.47, while the bivariate miss probability is *P*_M_(NFκB & ATF-2) = 0.42. A similar behavior is observed for the false alarm probabilities.

## 4. Conclusions

In this paper, we introduce and develop a set of statistical signal processing and decision-theoretic methods and metrics for modeling and measurement of decision-making errors in a TNF signaling system that regulates the two important transcription factors NFκB and ATF-2. As a useful and informative visual tool, first, optimal decision threshold curves (DTCs) are computed and graphed in the two-dimensional NFκB/ATF-2 plane (Figure 2D–F and Figure 3D–F), where an optimal DTC is a curve that divides the two-dimensional output space such that the error probability of making decisions on the TNF signal level is minimized.

Second, using measured nuclear NFκB and ATF-2 concentrations, false alarm (*P*_FA_) and miss (*P*_M_) error probabilities are computed. Here, *P*_FA_ is the error probability of deciding that TNF is high while it is indeed low, whereas *P*_M_ is the error probability of deciding that TNF is low, even though it is actually high. We observe a monotone decrease in both decision error rates in 30 min vs. the TNF signal level (Figure 4), perhaps because of the linear structure of the pathway in the short term. At 4 h, however, a monotone decrease in the decision error probabilities is not observed (Figure 4), possibly due to the activation of the A20 feedback that induces a nonlinear structure for the pathway. We also notice that *P*_FA_ and *P*_M_ increase from 30 min to 4 h (Figure 4A,B, respectively). This can be because of the reductions in the nuclear NFκB and ATF-2 concentrations in 4 h—due to the negative A20 feedback—that make the two heatmap clusters get closer to each other and overlap further (Figure 3), compared to the 30 min heatmap clusters’ overlap (Figure 2).

Third, we look at each system output alone to understand their relative individual importance in providing decisions on the status of the input TNF signal, compared to the decisions made using both outputs together. We observe that ATF-2 plays a smaller role compared to NFκB. This behavior of one output being less important than the other, however, may be specific to the signaling system studied here, and may not necessarily hold true for other signaling systems.

In conclusion, the developed statistical signal processing and decision-theoretic metrics and methods can quantify complex cellular decision-making processes and behaviors. The introduced metrics and methods can be applied to other and larger signaling systems that have several inputs, such as ligands or second messengers, and outputs, such as multiple transcription factors. More specifically, if the measured concentration levels of *N* molecules are available, then one can use the equations presented in Section 2 to compute the false alarm and miss error probabilities for cellular decisions. In this paper, the case of *N* = 2, a two-output system, is studied. This allowed us to compute and graph the optimal decision threshold curves in a two-dimensional plane to gain some useful insight. For *N* = 3, a three-output system, such graphical insight can still be obtained by computing and graphing the optimal decision threshold surfaces in a three-dimensional space using the equations presented in Section 2. If the number of output molecules of interest is greater than 3, *N* > 3, the cellular decision error probabilities can still be computed using the equations in Section 2. A direct visual inspection of the decision boundaries, however, is not feasible. In such scenarios, we can nevertheless look at the decision threshold curves for various pairs of molecules.

## Figures and Tables

**Table 1:** Importance scores of NFκB and ATF-2 for decision making in cells after 30 min and 4 h of TNF exposure.

Time	High TNF Level (ng/mL)	Importance Score	
		NFκB	ATF-2
30 min	0.082	0.18	0
	3.2	0.49	0.15
	50	0.57	0.2
4 h	0.082	0.05	0.01
	3.2	0.3	0.1
	50	0.24	0.07

## Data Availability

The data presented in this study are available upon request.
